# Gut microbiota and metabolic characteristics in subthreshold depression based on multi-omics

**DOI:** 10.3389/fpsyt.2026.1760479

**Published:** 2026-02-20

**Authors:** Jin Xian, Ling Wang, RuPing Shang, Mi Sun, Hui-Juan Yua, Xin Zhang, Bin Cheng, Shi-Jun Wang, Qi-Wen Tan

**Affiliations:** 1 Shandong University of Traditional Chinese Medicine, Jinan, Shandong, China; 2 Affiliated Hospital of Shandong University of Traditional Chinese Medicine, Jinan, Shandong, China

**Keywords:** gut brain axis, gut microbiota, lipid metabolism, microbiota–metabolite axis, subthreshold depression

## Abstract

**Background:**

Subthreshold depression (SD) is an intermediate state between normal mood and major depressive disorder (MDD), but its biological underpinnings remain insufficiently understood. Increasing evidence suggests that gut microbiota and host metabolic alterations may contribute to early depressive pathophysiology.

**Methods:**

We performed full-length 16S rRNA gene sequencing and LC–MS-based untargeted metabolomics on stool and plasma samples obtained from SD subjects and healthy controls. Microbial diversity, taxonomic composition, metabolic pathway alterations, and gut microbiota–metabolite associations were analyzed using bioinformatics pipelines, KEGG annotation, and Spearman correlation analysis.

**Results:**

SD patients exhibited marked gut microbial disturbances, including reduced microbial diversity and altered abundances of key genera such as decreased Eubacterium hallii group, Blautia, Dorea, and Agathobacter, and increased Escherichia–Shigella, Monoglobus, and Lachnoclostridium. Metabolomic profiling identified widespread metabolic perturbations, mainly affecting lipid metabolism, steroid hormone biosynthesis, and amino acid pathways. Exploratory correlation analysis indicated that beneficial taxa (e.g., Eubacterium hallii group and Blautia) were positively associated with specific glycerophospholipid and steroid hormone metabolites, whereas inverse associations were observed for other lipid-related metabolites.

**Conclusion:**

This integrative microbiome–metabolome analysis demonstrates that SD is accompanied by early disruptions in gut microbial composition and systemic metabolism, particularly within lipid-related pathways. These findings suggest that gut microbiota dysbiosis may reflect early metabolic dysregulation and depression-related biological vulnerability in SD and highlight the gut microbiota as a candidate biological target for early identification and intervention.

## Introduction

Subthreshold depression (SD) refers to individuals exhibiting depressive symptoms that do not meet the diagnostic criteria for major depressive disorder (MDD) (1). As a psychological subhealth state between normal mood and MDD, SD has received increasing clinical attention (2–5). Despite its prevalence, the pathogenesis of SD remains unclear, warranting further investigation.

The gut microbiota, consisting of bacteria, fungi, archaea, and viruses, has emerged as a critical factor in neuropsychiatric disorders. It contributes to host metabolism and neural regulation through the production of key metabolites such as dopamine, serotonin, tryptophan, and short-chain fatty acids (6). Dysbiosis of the gut microbiota has been linked to gastrointestinal disorders, including inflammatory bowel disease, and can affect brain function via immune modulation, the sympathetic nervous system, and microbial metabolites—collectively forming the gut–brain axis (7). Increasing evidence indicates a strong association between gut microbiota and depression (8–13). For instance, fecal microbiota transplantation from patients with depression induces depressive-like behaviors in mice, whereas transplantation from healthy individuals alleviates these behaviors (14). Dysbiosis-related increases in inflammatory cytokines and alterations in microbial metabolite concentrations may impair the integrity of both the gut and blood–brain barriers, leading to neuroinflammation (15). Although previous studies have highlighted the role of gut microbiota in depression, its mechanisms in SD remain largely unclear. Recent findings suggest that even mild depressive symptoms in SD are associated with disruptions in the gut microbiota, which may underlie its pathogenesis (4, 16). Understanding this relationship may provide novel strategies for the early prevention of MDD.

Metabolomics, which focuses on small-molecule metabolites, has substantially advanced research on depression. Alterations in tryptophan–kynurenine and fatty acid metabolic pathways, including reduced levels of L-tryptophan and kynurenine in MDD, have been identified (17). Integrating metabolomics with gut microbiota analysis provides an innovative approach for elucidating the mechanisms of SD.

Therefore, this study aimed to investigate the gut microbiota and plasma metabolomic profiles of individuals with SD. By analyzing compositional and metabolic alterations, we sought to elucidate the biological mechanisms underlying SD and lay a foundation for early diagnosis, intervention, and clinical advancement in this field.

## Methods

### Ethics statement

This study utilized a biobank established in our previous research (Registration No.: ChiCTR2100049660), which was approved by the Ethics Committee of the Affiliated Hospital of Shandong University of Traditional Chinese Medicine [(2021) Ethics Approval No. (055)-KY]. All procedures involving human participants were conducted in accordance with institutional ethical guidelines, and written informed consent was obtained from all participants prior to enrollment.

### Human population recruitment

Participants were recruited from the Affiliated Hospital of Shandong University of Traditional Chinese Medicine, including 64 individuals with SD and 32 healthy controls (HC). Initial screening was performed using the Patient Health Questionnaire-9 (PHQ-9), followed by the Mini International Neuropsychiatric Interview (MINI, version 5.0) to confirm depressive symptoms while ensuring that SD participants did not meet the DSM-V criteria for MDD. Inclusion criteria for the SD group were as follows: age 18–60 years; Hamilton Depression Rating Scale (HAMD-17) scores between 7 and 16; and no prior treatment for depression or use of antibiotics, probiotics, or fermented dairy products within the preceding two weeks. Exclusion criteria were diagnoses of MDD, bipolar disorder, or psychotic disorders; severe hepatic or renal dysfunction; serious arrhythmia or cardiac insufficiency; pregnancy; depression secondary to psychoactive substances or neurological diseases; suicidal ideation or attempts; or inability to comply with study procedures. HC participants were required to provide a recent medical check-up report and to be free of psychiatric disorders according to the MINI interview, and they were subjected to the same exclusion criteria as the SD group. Demographic and clinical information—including age, gender, body mass index (BMI), emotional status, exercise habits, and alcohol/tobacco use—was collected from all participants. Chi-square and Fisher’s exact tests revealed no significant between-group differences in gender distribution, age, BMI, emotional status, exercise frequency, or smoking behavior (**Supplementary Table 1**).

### Sample collection

Fresh stool samples (~1 g) were collected using sterile sampling spoons and transferred into pre-labeled 2-ml cryovials (Biologix, USA), which were immediately stored in liquid nitrogen until analysis. Participants fasted for at least 8 hours prior to blood sampling, and female participants avoided sample collection during menstruation. On the following morning (8:00–9:00 AM), approximately 4 ml of fasting venous blood was drawn into EDTA anticoagulant tubes. Samples were centrifuged at 3000 rpm for 10 minutes at 4 °C, and plasma was aliquoted into cryovials and stored at −80 °C for subsequent metabolomic analysis.

### DNA extraction and analysis methods for stool samples

Microbial DNA was extracted from stool samples using the E.Z.N.A.^®^ Soil DNA Kit (Omega Bio-tek, USA). The full-length 16S rRNA gene was amplified with primers 27F and 1492R (18) and sequenced on the PacBio Sequel II platform (Pacific Biosciences, USA) at Majorbio (Shanghai, China). CCS reads were processed with the DADA2-CCS plugin (19) in QIIME2 (20). Samples were rarefied to 6,000 reads, achieving an average Good’s coverage of 99.09%. Amplicon sequence variants (ASVs) were assigned using the SILVA v138 database. Alpha diversity metrics, including ACE, Chao, Shannon, Simpson, Shannon evenness, and Simpson evenness indices, were calculated to evaluate microbial diversity, and inter-group differences were analyzed using the Wilcoxon test. Beta diversity was assessed using Principal Coordinates Analysis (PCoA) with weighted and unweighted UniFrac distances, and group differences were tested with the Adonis (PERMANOVA) test. Spearman correlations (|r| > 0.2, P < 0.05) were used to identify genera for network analysis (21). Statistical analyses were performed in R 4.3.1 and the Majorbio Cloud Platform (22).

### Metabolite profiling and data processing for plasma samples

Plasma metabolites were extracted using a precooled extraction solution and processed by vortexing, ultrasonic-assisted extraction, and centrifugation. The resulting supernatants were analyzed using ultra-high-performance liquid chromatography–mass spectrometry (UHPLC–MS). Raw data were processed with Progenesis QI (Waters Corporation, USA) and matched against HMDB, Metlin, and the Majorbio in-house database for metabolite identification. Features detected in <80% of samples were removed, and peak intensities were normalized. Variables with a relative standard deviation (RSD) >30% in quality control samples were excluded, and data were log10-transformed before statistical analysis. Multivariate analysis was conducted using OPLS–DA in the ropls R package (version 1.6.2), complemented by Wilcoxon rank-sum tests and fold-change analysis. Metabolites were considered significantly altered when meeting both criteria: VIP >1 and p < 0.05. Differential metabolites were mapped to biochemical pathways through KEGG-based enrichment and pathway topology analyses, and categorized according to pathway and functional classes.

### Statistical analysis

The identified endogenous differential metabolites were treated as environmental variables and correlated with the top 30 bacterial genera ranked by relative abundance. Associations between bacterial genera and metabolic features were evaluated using Spearman correlation coefficients.

## Results

### Gut microbiota changes in SD

**Figure 1:**
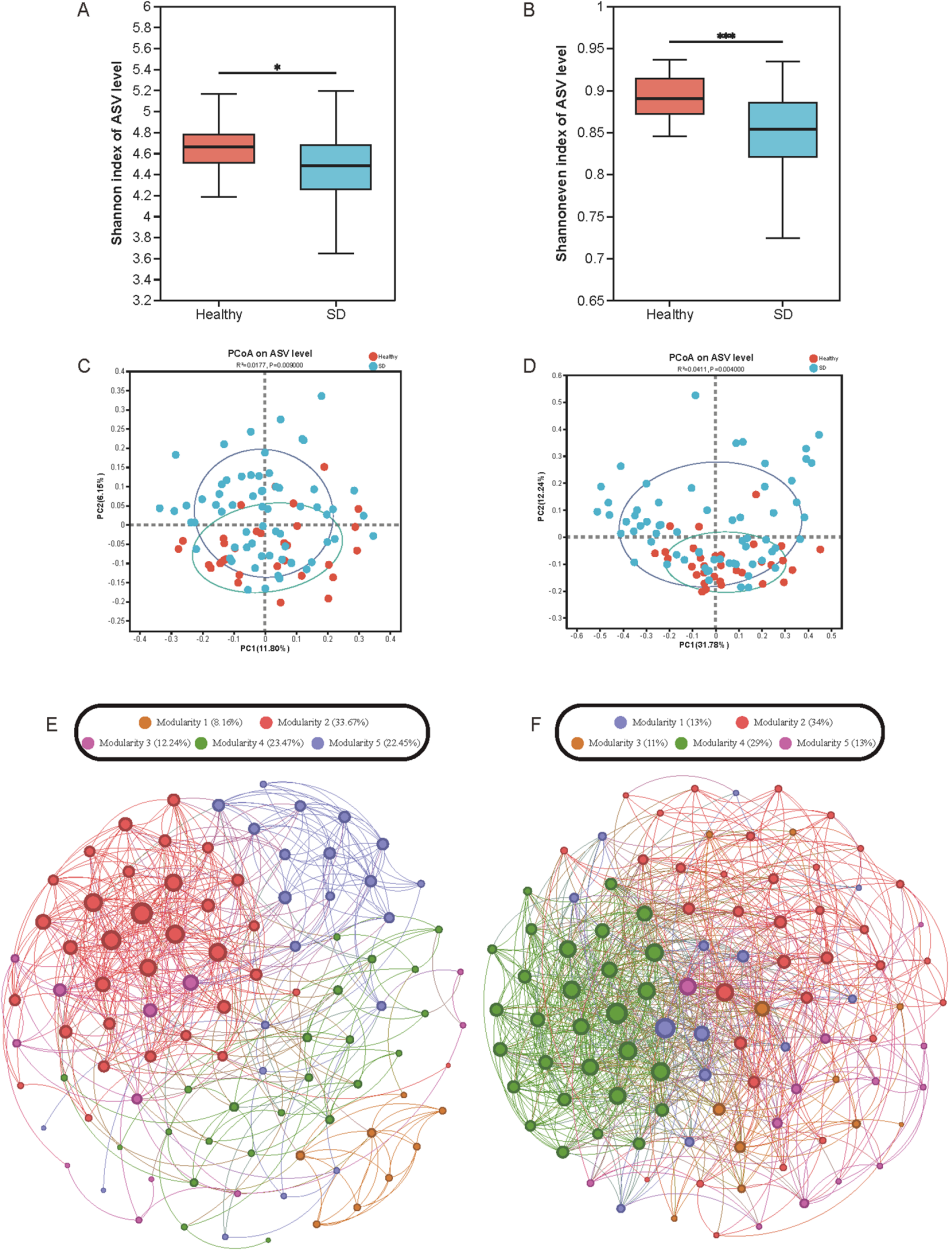
**(A, B)** Wilcoxon rank-sum test for Shannon and shannoneven index; **(B, C)** PCoA on ASV level based on unweighted_unifrac and weighted_unifrac; **(D, E)** Correlation network between SD and healthy individuals. *0.01 < P ≤ 0.05, ***P ≤ 0.001.

To characterize the gut microbiota features of individuals with SD, full-length 16S rRNA sequencing was performed on 96 fecal samples. After sequence denoising, a total of 11,184 ASVs and 1,804,258 high-quality reads were obtained, with an average of 18,794 reads per sample. The core ASV accumulation curve reached a plateau (**Supplementary Figure S1A**), indicating that the sample size was adequate. Rarefaction curves approached saturation (**Supplementary Figures S1B, C**), suggesting sufficient sequencing depth. At the ASV level, no significant differences were observed in richness indices, including Chao1 and ACE (**Supplementary Figures S1D, E**). However, the SD group exhibited significantly lower Shannon and Shannon evenness indices compared with the HC group (P < 0.05 and P < 0.01, respectively; **Figures 1A, B**), reflecting reduced gut microbial diversity and community evenness. Principal coordinate analysis (PCoA) based on weighted and unweighted UniFrac distances further revealed significant separation between the SD and HC groups (both P < 0.01; **Figures 1C, D**). Similar clustering patterns were observed at the genus level (**Supplementary Figures S1F. G**). Correlation network and module analyses showed that the HC microbiota network tended to be scale-free with fewer inter-module connections, whereas the SD network was more complex, characterized by increased connectivity within and between modules (**Figures 1E, F**). These results collectively demonstrate pronounced alterations in gut microbiota composition and structure in individuals with SD compared with HC.

**Figure 2:**
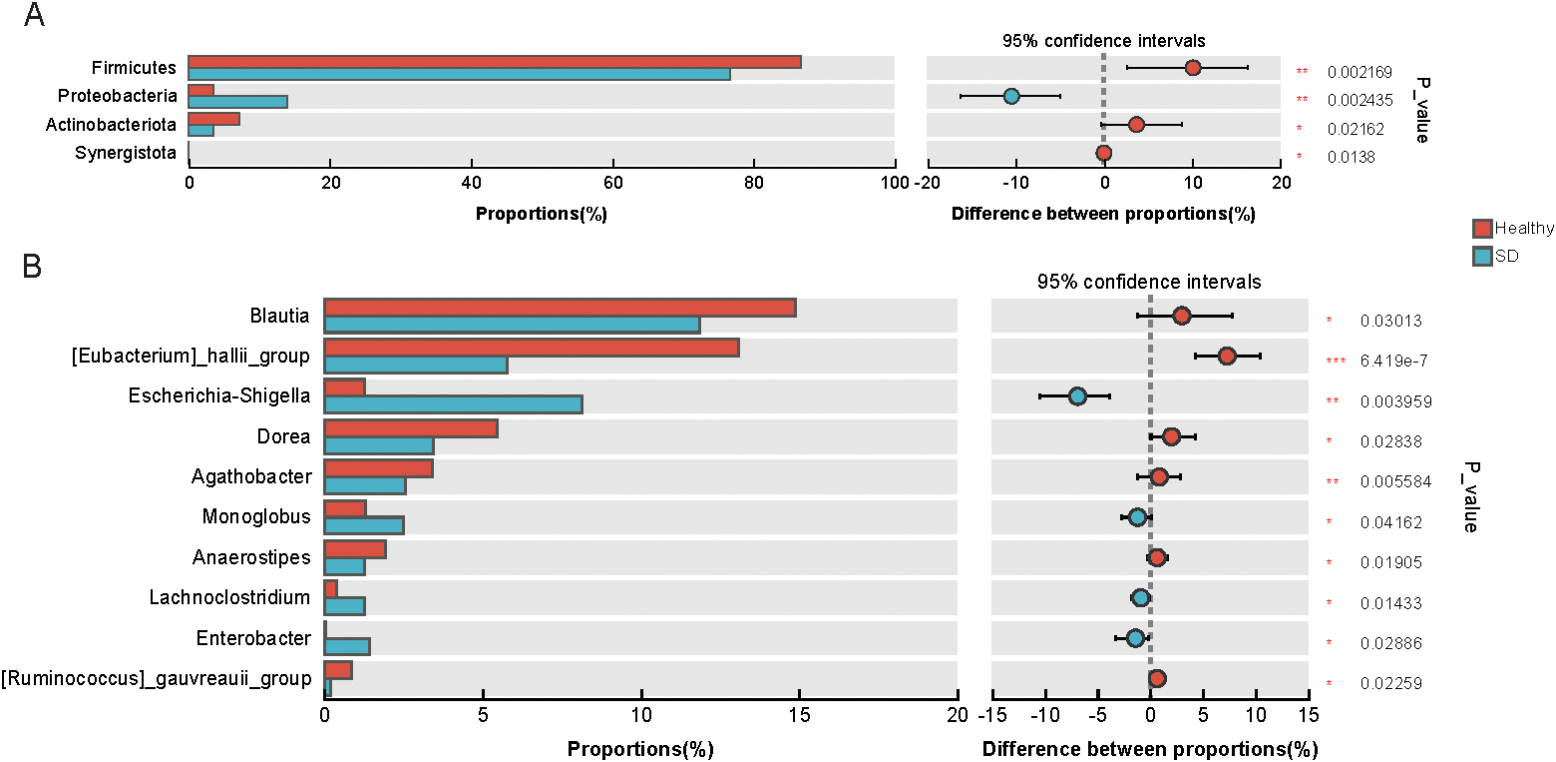
**(A, B)** The significantly different phyla/genera between SD and healthy. *0.01 < P ≤ 0.05, **0.001 < P ≤ 0.01, ***P ≤ 0.001.

Further differential abundance analysis revealed marked alterations in the gut microbiota between the SD and HC groups. Compared with the HC group, the SD group showed significant decreases in Firmicutes and Actinobacteriota, together with significant increases in Proteobacteria and Synergistota (**Figure 2A**). At the genus level, the SD group exhibited significantly lower abundances of *Blautia*, *Eubacterium* hallii group, *Dorea*, *Agathobacter*, *Anaerostipes*, and *Ruminococcus* gauvreauii group, whereas Escherichia–Shigella, *Monoglobus*, *Lachnoclostridium*, and *Enterobacter* were significantly enriched (**Figure 2B**). These results were further supported by LEfSe analysis (**Supplementary Figure S1H**). Taken together, these findings indicate that although depressive symptoms in SD individuals are relatively mild, distinct alterations in gut microbiota composition are already evident, suggesting a potential involvement of gut dysbiosis in the early stages of depression.

### Plasma metabolic profiling reveals apparent discrepancy between SD and HC

**Figure 3:**
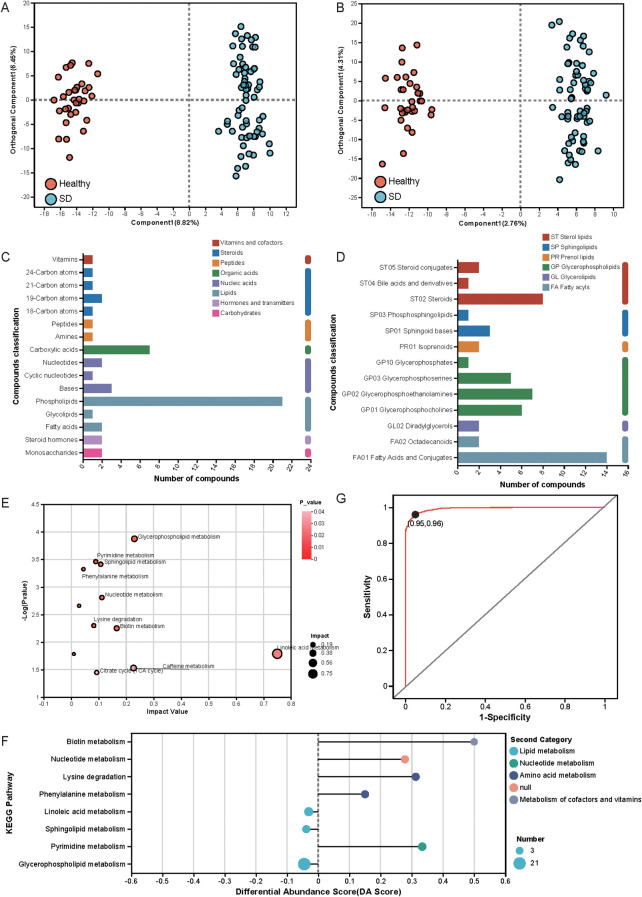
**(A, B)** OPLS-DA of plasma metabolic profiles in SD and healthy controls under positive and negative ion modes; **(C)** KEGG compound classification based on compounds with biological roles; **(D)** KEGG compound classification based on lipids; **(E)** KEGG topology analysis; **(F)** Differential abundance score plot; **(G)** ROC curve constructed from three lipid metabolism-related plasma metabolites.

OPLS-DA analysis demonstrated clear separation between the plasma metabolic profiles of individuals with SD and HC (**Figures 3A, B**), and the robustness of the model was supported by OPLS-DA permutation testing (**Supplementary Figures S2A, B**). These results indicate substantial differences in plasma metabolic phenotypes between the two groups. Based on P < 0.05 and VIP ≥ 1, a total of 615 differential metabolites were identified.

To explore the biological significance of these SD-related metabolites, we performed KEGG annotation and obtained 112 annotated plasma metabolites (**Supplementary Table S2**). KEGG compound classification showed that 49 metabolites mapped to eight biologically relevant categories, with lipids being the most abundant, followed by organic acids (**Figure 3C**). Furthermore, 54 compounds were assigned to six lipid subclasses, with glycerophospholipids being the most prevalent, followed by fatty acyls, sterol lipids, sphingolipids, prenol lipids, and glycerolipids (**Figure 3D**). KEGG pathway topology analysis revealed enrichment of several key metabolic pathways, including linoleic acid metabolism, glycerophospholipid metabolism, caffeine metabolism, biotin metabolism, sphingolipid metabolism, nucleotide metabolism, the citrate (TCA) cycle, pyrimidine metabolism, lysine degradation, and phenylalanine metabolism (**Figure 3E**, **Supplementary Table S3**). Eight pathways exhibited significant differences: biotin metabolism, nucleotide metabolism, lysine degradation, phenylalanine metabolism, and pyrimidine metabolism were elevated overall, while linoleic acid metabolism, glycerophospholipid metabolism, and sphingolipid metabolism showed overall decreases (**Figure 3F**).

To assess the diagnostic potential of the identified metabolites, ROC curve analysis was performed. Eight endogenous metabolites had an AUC greater than 0.9 (**Supplementary Figures S2 C–I**, **Supplementary Table S4**), including three involved in lipid metabolism. The combined ROC curve for these metabolites produced an AUC of 0.993, with 96% sensitivity and 95% specificity (**Figure 3G**), indicating that this metabolite panel may serve as a promising biomarker set for the clinical identification of SD.

### Correlation analysis of plasma metabolome and gut microbiota

**Figure 4:**
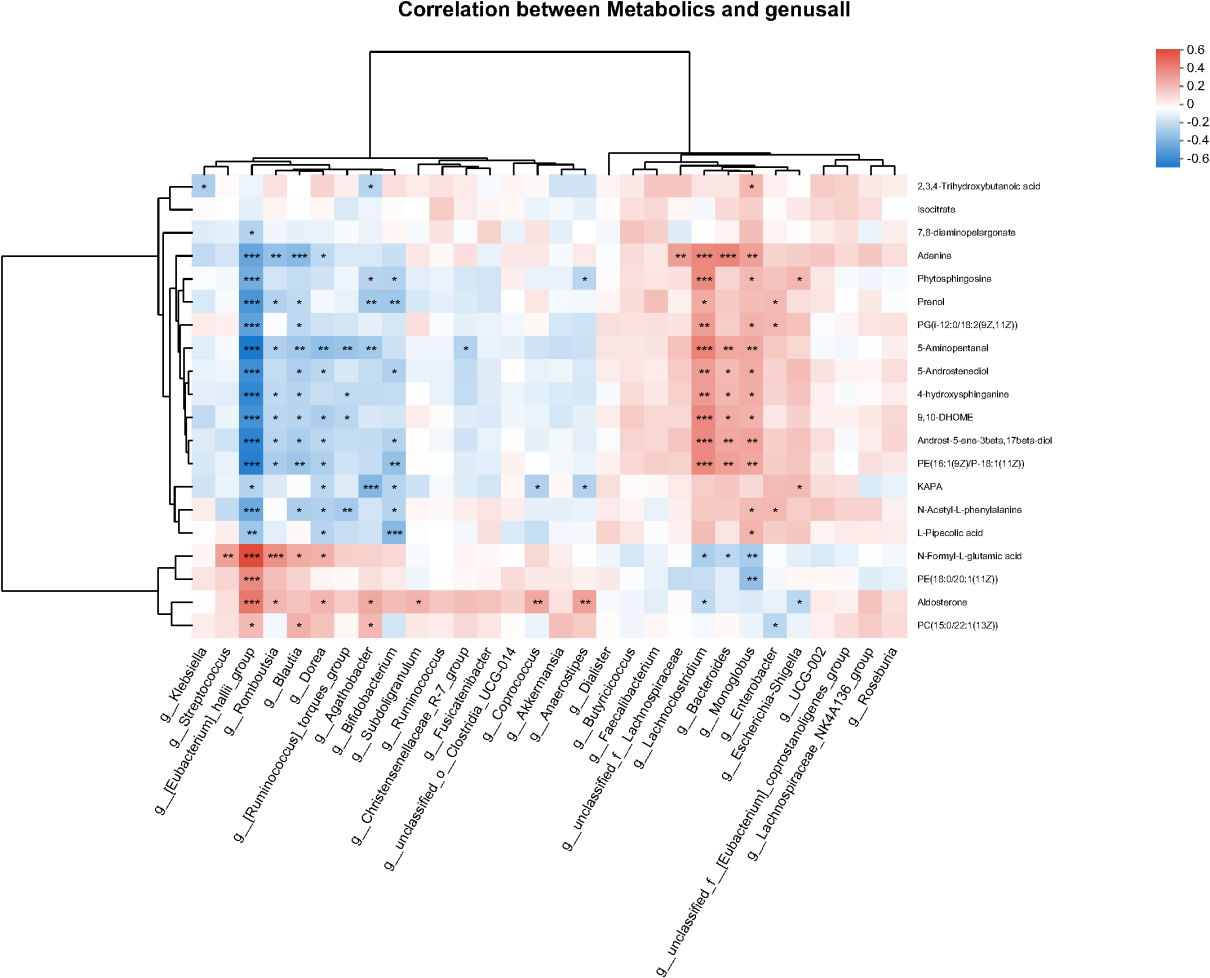
Heatmap of correlations between gut microbiota and SD-related differential metabolites in plasma. *0.01 < P ≤ 0.05, **0.001 < P ≤ 0.01, ***P ≤ 0.001.

The most important differential metabolites were first identified using random forest analysis (**Supplementary Table S5**), followed by Spearman correlation analysis to assess associations between plasma metabolites and gut microbiota. The results showed that *Eubacterium* hallii group, *Blautia*, *Dorea*, and *Agathobacter*, which were reduced in abundance in SD subjects, were significantly positively correlated with decreased plasma levels of N-Formyl-L-glutamic acid, PE[18:0/20:1(11Z)], Aldosterone, and PC[15:0/22:1(13Z)], and negatively correlated with several upregulated plasma metabolites. In healthy individuals, Lachnospiraceae and Escherichia–Shigella, which were also lower in abundance, displayed the opposite correlation pattern with plasma metabolites (**Figure 4**).

Androst-5-ene-3β,17β-diol, 5-Androstenediol, and Aldosterone are metabolites involved in steroid hormone biosynthesis. PE[16:1(9Z)/P-18:1(11Z)], PE[18:0/20:1(11Z)], PG[i-12:0/18:2(9Z,11Z)], and PC(15:0/22:1(13Z)) participate in glycerophospholipid metabolism, whereas Phytosphingosine and 4-hydroxysphinganine are components of sphingolipid metabolism. These findings suggest coordinated alterations between gut microbiota and host metabolic pathways in SD, particularly those related to lipid metabolism.

## Discussion

To our knowledge, this study is the first to integrate full-length 16S rRNA sequencing with LC–MS-based untargeted metabolomics to characterize both gut microbial composition and plasma metabolic profiles in individuals with SD. Although SD does not meet the diagnostic threshold for MDD, our findings demonstrate that measurable biological perturbations—in both gut microbiota and circulating metabolites—are already present, supporting the conceptualization of SD as a potential risk state preceding MDD (23).

At the microbial community level, SD was characterized by reduced diversity and evenness but preserved richness, a pattern partly consistent with observations in MDD (24). However, the magnitude of change appeared less pronounced, suggesting that SD may represent an early or intermediate stage of microbiome disruption along the depression continuum. Network analysis further supported this notion: the microbial network in HC exhibited a scale-free topology with limited inter-module connectivity, indicative of stable ecological organization. In contrast, SD showed more complex and densely interconnected networks, implying greater ecological instability and more frequent microbial interaction dynamics. Such instability may predispose individuals to further dysbiosis under stress or environmental challenges.

Taxonomic differences were also consistent with known microbial signatures of depression. Reductions in Firmicutes and Actinobacteriota, together with increases in Proteobacteria and Synergistota, indicate a shift from beneficial taxa toward potential pathobionts. Members of Firmicutes synthesize key neuroactive metabolites—including glutamate, butyrate, serotonin, and γ-aminobutyric acid (GABA)—that are implicated in MDD pathophysiology (25), and decreased Firmicutes abundance has been observed in drug-naive MDD patients (26). Although Actinobacteriota represent a smaller fraction of the microbiota, they contribute to gut homeostasis, and their reported increase in MDD (27) indicates that phylum-level changes may vary by disease stage or host characteristics. By contrast, the enrichment of Proteobacteria—commonly associated with inflammatory and metabolic disturbances—in SD is notable, as this phylum has been positively associated with psychological distress, anxiety, and depressive symptoms (28, 29).

At the genus level, SD was marked by reduced abundances of Blautia, Eubacterium hallii group, Dorea, and Agathobacter, accompanied by increases in Escherichia–Shigella, Monoglobus, and Lachnoclostridium. These genera are strongly associated with mental health: Blautia abundance correlates inversely with depression severity (30), and Eubacterium hallii, a major short-chain fatty acid (SCFA) producer, contributes to gut barrier integrity and anti-inflammatory signaling. Conversely, Escherichia–Shigella is a pro-inflammatory taxon associated not only with diarrheal disease but also with lipid metabolic disturbances (31), suggesting a potential mechanistic link to metabolic alterations observed in SD. Collectively, these findings highlight a microbiota profile in SD characterized by decreased beneficial bacteria and increased potential pathogens—changes directionally consistent with MDD but milder in magnitude.

The metabolomic results further reveal widespread systemic alterations in SD. A total of 112 annotated metabolites differed between groups, with 84 upregulated and 28 downregulated. The predominance of lipid-related compounds among the differential metabolites suggests that lipid metabolism may be particularly sensitive to early depressive states. Lipids play essential roles in inflammation, neurotransmission, membrane structure, and hormone synthesis. Previous studies have linked lipid dysregulation to obesity, type 2 diabetes, non-alcoholic fatty liver disease, cancer, and cardiovascular conditions (32), but accumulating evidence also implicates lipid metabolism in depression through mechanisms involving acidic sphingomyelinase activation, phospholipase A2 signaling, oxidative stress, and lipid peroxidation (33). Altered cholesterol metabolism has similarly been associated with anxiety and depression (34).

KEGG pathway analysis further identified several functionally important pathways, including upregulated lysine degradation, phenylalanine metabolism, biotin metabolism, nucleotide metabolism, and pyrimidine metabolism, alongside downregulated linoleic acid, glycerophospholipid, and sphingolipid metabolism. The amino acid pathways identified—particularly lysine and phenylalanine—are relevant to mitochondrial energy regulation and epigenetic modulation (35–37), both implicated in depression. Nucleotide and pyrimidine metabolism pathways, involving purinergic receptors such as P2X7 expressed on neurons and microglia (38), play important roles in synaptic plasticity and inflammation (39), suggesting that these pathways may participate in early depressive pathophysiology. Among lipid pathways, the consistent downregulation of phosphatidylcholine (PC) in linoleic acid and glycerophospholipid metabolism aligns with reports that reductions in PCs and glycerophospholipids are associated with depression onset (33, 40). Sphingolipid metabolism also emerged as relevant: elevated acidic sphingomyelinase (ASM) activity and ceramide accumulation have been documented in depression and attenuated by antidepressants (41–43), indicating that sphingolipid dysregulation may serve as an early biochemical marker of SD.

Our integrative microbiome–metabolome correlation analysis suggests that the gut microbiota may modulate lipid and steroid hormone pathways in SD. Beneficial genera such as *Eubacterium* hallii group and *Blautia* showed positive correlations with key metabolites in glycerophospholipid metabolism and steroid hormone biosynthesis, whereas opposing patterns were observed for taxa enriched in SD. Notably, the correlation patterns we observed differ from those typically reported in MDD (44), possibly reflecting preserved compensatory metabolic mechanisms in SD despite detectable imbalances. Together, these findings support a model in which early alterations in gut microbial composition influence host lipid, amino acid, and steroid hormone metabolism, contributing to the metabolic phenotype characteristic of SD. Notably, these microbiota and metabolic alterations were observed despite relatively mild clinical symptoms, suggesting that they may represent state-related biological features of SD rather than markers directly reflecting symptom severity. The opposite directions observed across lipid-related metabolites may indicate lipid class–specific regulation and compensatory remodeling in early depressive states. This warrants follow-up studies using targeted lipidomics with absolute quantification and longitudinal sampling to validate and track microbiota-linked lipid changes in SD.

This study has several limitations. First, participants were all Han Chinese from a single geographic region with similar dietary habits; although meal regularity was recorded, detailed dietary intake data were not collected, which may affect microbiome and metabolomic profiles. Second, the relatively modest sample size may limit generalizability. Third, full-length 16S rRNA sequencing offers limited resolution at the species level compared with metagenomic approaches. Finally, our findings are observational and require validation through mechanistic studies, including animal models. Future research should include larger and more diverse cohorts, integrate metagenomic and multi-omics analyses, and explore dietary and environmental modulators to better elucidate the microbiota–metabolite–brain axis in SD.

## Conclusion

This study demonstrates that individuals with SD exhibit clear disturbances in both gut microbiota composition and plasma metabolic profiles, despite not meeting the diagnostic threshold for MDD. The coordinated alterations in lipid metabolism, steroid hormone biosynthesis, and amino acid pathways, together with shifts in key microbial taxa, suggest an early disruption of the microbiota–metabolite axis that may contribute to depression-related biological vulnerability and metabolic dysregulation. These findings highlight the potential value of gut microbiota as a candidate biological target for early identification and intervention in SD. Nevertheless, further mechanistic, and longitudinal studies are needed to clarify causal pathways and evaluate whether microbiota-based strategies can prevent progression from SD to MDD.

## Data Availability

The original contributions presented in the study are included in the article/**Supplementary Material**. Further inquiries can be directed to the corresponding authors.
