# Supplementary material for: Gut microbiota and metabolic characteristics in subthreshold depression based on multi-omics
Source: Front Psychiatry. 2026 Feb 20;17:1760479. doi: 10.3389/fpsyt.2026.1760479 (PMC12963311; doi:10.3389/fpsyt.2026.1760479)
Supplement: Supplementary file 1 [file SupplementaryFile1.docx]

**Figure S1 Gut Microbial Diversity and Community Structure Analysis in Subthreshold Depression and Healthy Controls**


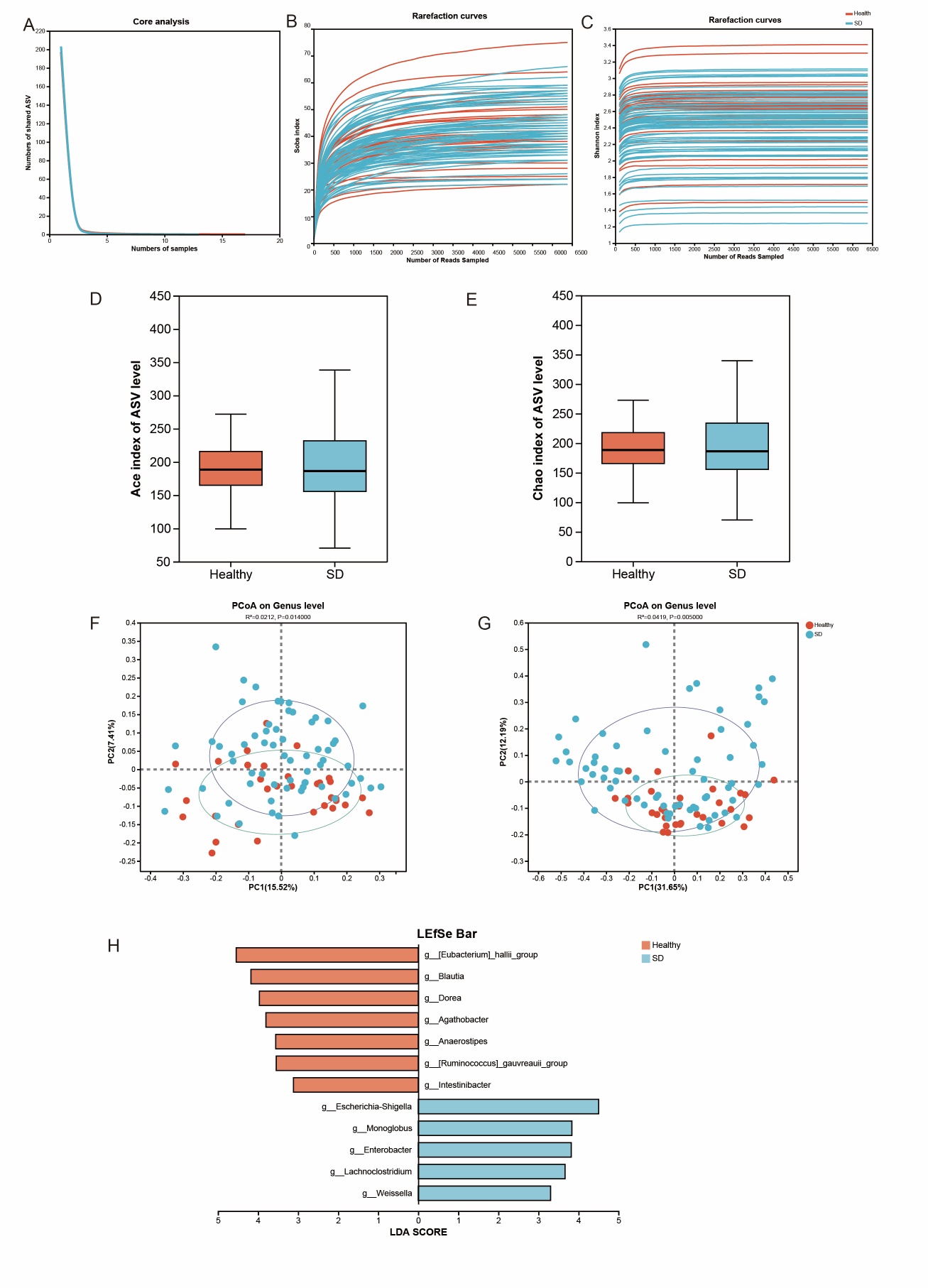


Figure S1 A Core ASV analysis in SD and healthy; B Rarefaction curves of all samples; C Shannon curves of all samples; D-E Wilcoxon rank-sum test for ace index and chao index; F-G PCoA on Genus level base on unweighted_unifrac and weighted_unifrac; H LEfSe Bar

**Figure S2 OPLS-DA and Biomarker Analysis of Plasma Metabolites in Subthreshold Depression and Healthy Individuals**


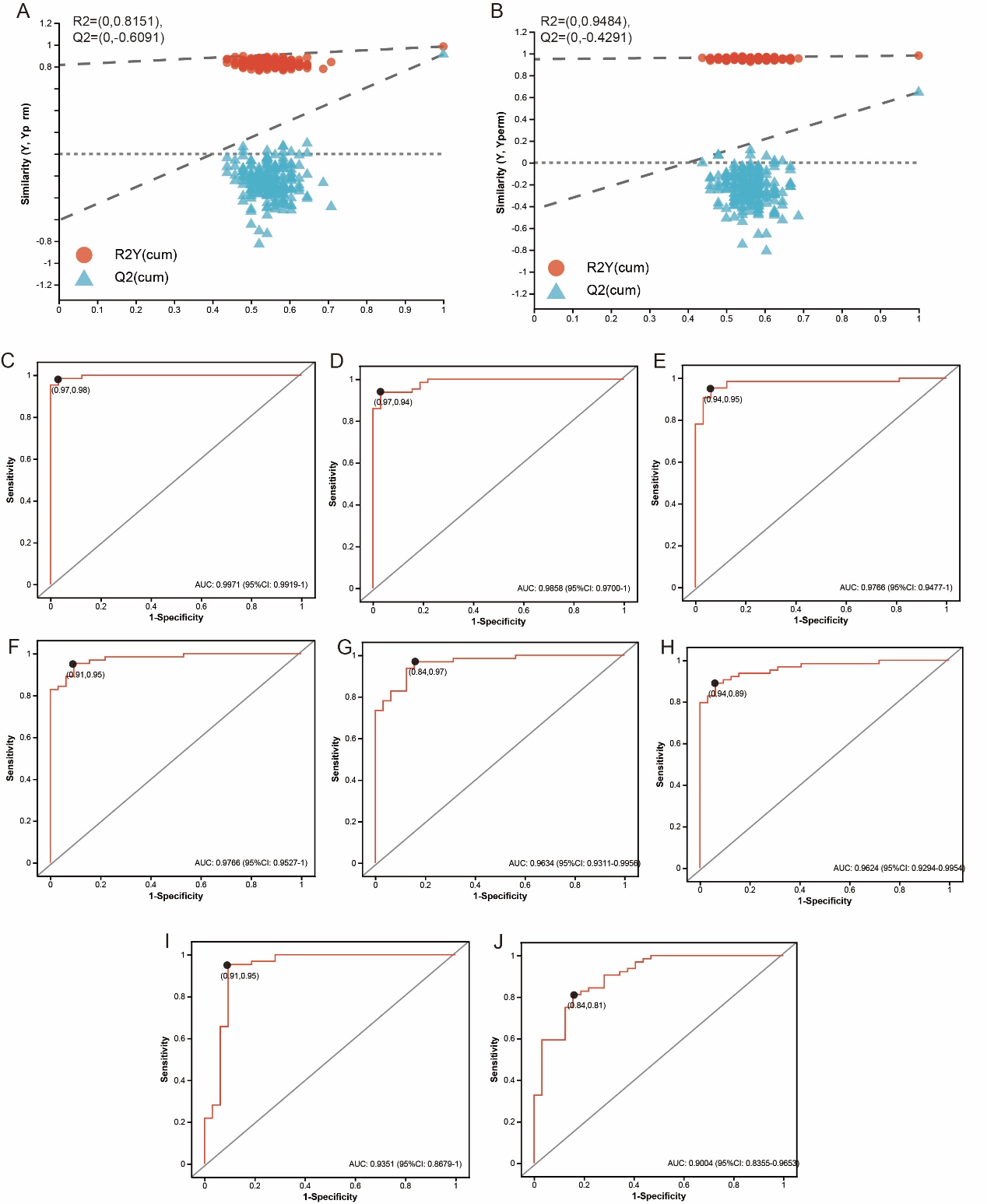


Figure S2 A-B OPLS-DA permutation test plot of plasma metabolic profiles in SD and healthy controls under positive and negative ion modes; C-I ROC curves of 8 plasma biomarkers (AUC > 0.9), including Androst-5-ene-3β,17β-diol, Phytosphingosine, 4-Hydroxysphinganine, PE(16:1(9Z)/P-18:1(11Z)), 5-Aminopentanal, 5-Androstenediol, KAPA, and Prenol.

**Table S1 Clinical Characteristics of Subjects**

|  | **HC** | **SD** | *P* |
| --- | --- | --- | --- |
| Gender, n (%): |  |  | 1.000 |
| Male | 9 （28.1%） | 18 （28.1%） |  |
| Female | 23 （71.9%） | 46 （71.9%） |  |
| Age, n (%): |  |  | 0.855 |
| 18-30 | 20 （62.5%） | 35 （54.7%） |  |
| 30-40 | 9 （28.1%） | 22 （34.4%） |  |
| 40-50 | 3 （9.38%） | 7 （10.9%） |  |
| BMI (Mean ± SD, kg/m²) | 21.7 ±2.90 | 22.6 ±3.17 | 0.175 |
| Emotional status, n (%): |  |  | 0.154 |
| Single | 12 （37.5%） | 30 （46.9%） |  |
| Divorced | 0 （0.00%） | 3 （4.69%） |  |
| In a relationship | 9 （28.1%） | 7 （10.9%） |  |
| Married | 11 （34.4%） | 24 （37.5%） |  |
| Exercise habits, n (%): |  |  | 0.263 |
| Regular | 8 （25.0%） | 8 （12.5%） |  |
| Occasional (1-2 times per week) | 14 （43.8%） | 29 （45.3%） |  |
| Regular (≥3 times per week) | 10 （31.2%） | 27 （42.2%） |  |
| Dietary habits, n (%): |  |  | 0.032 |
| Irregular time and amount | 2 （6.25%） | 18 （28.1%） |  |
| Fixed amount, irregular time | 2 （6.25%） | 8 （12.5%） |  |
| Fixed time, irregular amount | 10 （31.2%） | 15 （23.4%） |  |
| Regular time and amount | 18 （56.2%） | 23 （35.9%） |  |
| Smoking, n (%): |  |  | 0.643 |
| Non-smoker | 30 （93.8%） | 60 （93.8%） |  |
| Occasional (≤5 days per month) | 0 （0.00%） | 2 （3.12%） |  |
| Frequent (>6 days per month) | 2 （6.25%） | 2 （3.12%） |  |
| Alcohol consumption, n (%): |  |  | 0.036 |
| Non-drinker | 27 （84.4%） | 39 （60.9%） |  |
| Occasional (≤5 days per month) | 5 （15.6%） | 25 （39.1%） |  |
| HAMD (Mean ± SD) | - | 11.9±2.53 | - |
| PHQ-9 [M (Q25, Q75)] | 1.00 （0.75,2.00） | 10.0 （9.00,12.0） | <0.001 |
| GAD-7 [M (Q25, Q75)] | 0.00 （0.00,1.00） | 6.00 （5.00,8.25） | <0.001 |
| PCS[M（Q25,Q75）] | 56.9 （54.6,57.1） | 51.9 （48.7,56.2） | 0.001 |
| MCS[M（Q25,Q75）] | 57.4 （54.6,57.5） | 32.3 （27.7,39.6） | <0.001 |

**Table S2 Differential plasma metabolites between SD and healthy individuals**

| Metabolite | Regulate | KEGG Compound ID | Formula | VIP_pred_OPLS-DA | FC(SD/Healthy) | P_value | FDR |
| --- | --- | --- | --- | --- | --- | --- | --- |
| 2-Chloromaleylacetate | up | C06329 | C6H5ClO5 | 3.59 | 1.25 | 0.00 | 0.02 |
| L-Pipecolic acid | up | C00408 | C6H11NO2 | 3.47 | 1.39 | 6.20E-06 | 2.36E-05 |
| Malonic Acid | up | C00383 | C3H4O4 | 3.02 | 1.12 | 0.00 | 0.00 |
| 6-(alpha-D-Glucosaminyl)-1D-myo-inositol | up | C15658 | C12H23NO10 | 2.93 | 1.11 | 0.00 | 0.01 |
| 3-Methyldioxyindole | up | C05834 | C9H9NO2 | 2.92 | 1.27 | 8.74E-08 | 4.84E-07 |
| DG(20:3(5Z,8Z,11Z)/15:0/0:0) | up | C00165 | C38H68O5 | 2.70 | 1.08 | 0.00 | 0.02 |
| Lactosamine | up | C00203 | C12H23NO10 | 2.49 | 1.09 | 0.00 | 0.01 |
| 3,4-dihydroxyphenylacetic Acid | up | C01161 | C8H8O4 | 2.40 | 1.06 | 0.00 | 0.01 |
| Imidazoleacetic acid ribotide | up | C04437 | C10H15N2O9P | 2.23 | 1.05 | 0.00 | 0.01 |
| Beta-Alanyl-L-histidine | up | C00386 | C9H14N4O3 | 2.21 | 1.11 | 0.01 | 0.06 |
| 3,4-Dihydroxymandelic Acid | up | C05580 | C8H8O5 | 2.12 | 1.08 | 0.01 | 0.05 |
| Formyl-5-hydroxykynurenamine | up | C05647 | C10H12N2O3 | 1.99 | 1.05 | 0.00 | 0.03 |
| N-hydroxyl-tryptamine | up | C17203 | C10H12N2O | 1.97 | 1.06 | 0.00 | 0.01 |
| DG(18:3(9Z,12Z,15Z)/15:0/0:0) | up | C00165 | C36H64O5 | 1.92 | 1.08 | 0.00 | 0.00 |
| PE(16:1(9Z)/P-18:1(11Z)) | up | C00350 | C39H74NO7P | 1.92 | 1.03 | 3.41E-14 | 1.35E-12 |
| N-Acetyl-L-phenylalanine | up | C03519 | C11H13NO3 | 1.86 | 1.06 | 5.20E-09 | 3.62E-08 |
| Hydroquinone | up | C00530 | C6H6O2 | 1.85 | 1.09 | 4.00E-08 | 2.37E-07 |
| Epsilon-caprolactam | up | C06593 | C6H11NO | 1.84 | 1.03 | 2.75E-05 | 0.00 |
| 5-Acetamidovalerate | up | C03087 | C7H13NO3 | 1.82 | 1.03 | 1.19E-05 | 0.00 |
| Acetaminophen | up | C06804 | C8H9NO2 | 1.81 | 1.08 | 2.39E-13 | 5.56E-12 |
| 15-Hydroxynorandrostene-3,17-dione glucuronide | up | C03033 | C24H32O9 | 1.76 | 1.04 | 0.01 | 0.04 |
| Cytosine | up | C00380 | C4H5N3O | 1.74 | 1.05 | 0.00 | 0.02 |
| Sarcosine | up | C00213 | C3H7NO2 | 1.71 | 1.07 | 3.05E-05 | 0.00 |
| 5-Aminopentanal | up | C12455 | C5H11NO | 1.70 | 1.04 | 1.68E-13 | 4.31E-12 |
| Prenol | up | C01390 | C5H10O | 1.69 | 1.02 | 1.90E-10 | 3.58E-08 |
| (3S)-3,6-Diaminohexanoate | up | C01142 | C6H14N2O2 | 1.69 | 1.05 | 1.36E-06 | 5.86E-06 |
| 5-Androstenediol | up | C04295 | C19H30O2 | 1.69 | 1.04 | 1.89E-13 | 4.58E-12 |
| Adenine | up | C00147 | C5H5N5 | 1.69 | 1.03 | 4.76E-08 | 2.62E-06 |
| Dehydroepiandrosterone Sulfate | up | C04555 | C19H28O5S | 1.67 | 1.03 | 0.00 | 0.03 |
| PA(8:0/i-21:0) | up | C00416 | C32H63O8P | 1.65 | 1.13 | 0.00 | 0.00 |
| Dihydrocortisol | up | C05471 | C21H32O5 | 1.61 | 1.04 | 0.00 | 0.03 |
| 4-Hydroxybutyric acid | up | C00989 | C4H8O3 | 1.53 | 1.02 | 0.00 | 0.01 |
| Tetrahydrocortisol | up | C05472 | C21H34O5 | 1.53 | 1.03 | 0.05 | 0.16 |
| Androst-5-ene-3beta,17beta-diol | up | C04295 | C19H30O2 | 1.52 | 1.02 | 2.61E-15 | 4.65E-13 |
| Dihydrocorticosterone | up | C05475 | C21H32O4 | 1.52 | 1.05 | 0.03 | 0.11 |
| Vanillylamine | up | C16666 | C8H11NO2 | 1.52 | 1.04 | 0.00 | 0.00 |
| 13(S)-HODE | up | C14762 | C18H32O3 | 1.48 | 1.05 | 0.00 | 0.00 |
| Hydrocinnamic acid | up | C05629 | C9H10O2 | 1.48 | 1.07 | 0.03 | 0.12 |
| N-Acetyl-D-phenylalanine | up | C05620 | C11H13NO3 | 1.47 | 1.02 | 0.00 | 0.00 |
| Resiniferatoxin | up | C09179 | C37H40O9 | 1.47 | 1.02 | 0.01 | 0.06 |
| 4-hydroxysphinganine | up | C12144 | C18H39NO3 | 1.46 | 1.02 | 3.41E-14 | 1.35E-12 |
| Pimelic Acid | up | C02656 | C7H12O4 | 1.43 | 1.03 | 0.01 | 0.04 |
| 2,3,4-Trihydroxybutanoic acid | up | C01620 | C4H8O5 | 1.42 | 1.01 | 1.96E-07 | 8.53E-06 |
| 3-(3-Indolyl)-2-oxopropanoic acid | up | C00331 | C11H9NO3 | 1.39 | 1.02 | 0.00 | 0.02 |
| PG(i-12:0/18:2(9Z,11Z)) | up | C00344 | C36H67O10P | 1.39 | 1.04 | 7.75E-05 | 0.00 |
| 3-Dehydroquinic acid | up | C00944 | C7H10O6 | 1.35 | 1.02 | 0.00 | 0.03 |
| 17alpha-Estradiol | up | C02537 | C18H24O2 | 1.33 | 1.05 | 0.00 | 0.01 |
| 3,4-Dihydroxyhydrocinnamic acid | up | C10447 | C9H10O4 | 1.32 | 1.10 | 0.01 | 0.02 |
| D-Ornithine | up | C00515 | C5H12N2O2 | 1.32 | 1.04 | 0.00 | 0.00 |
| Phytosphingosine | up | C12144 | C18H39NO3 | 1.32 | 1.01 | 1.08E-14 | 7.31E-13 |
| 9-Oxo-nonanoic acid | up | C16322 | C9H16O3 | 1.29 | 1.04 | 0.03 | 0.11 |
| 2-Hydroxybutyric Acid | up | C05984 | C4H8O3 | 1.29 | 1.01 | 0.00 | 0.02 |
| Pantothenic Acid | up | C00864 | C9H17NO5 | 1.26 | 1.02 | 0.02 | 0.07 |
| Isocitrate | up | C00311 | C6H8O7 | 1.21 | 1.01 | 0.01 | 0.04 |
| 2-Methylhippuric Acid | up | C01586 | C10H11NO3 | 1.20 | 1.03 | 0.00 | 0.01 |
| Isoproterenol | up | C07056 | C11H17NO3 | 1.20 | 1.01 | 1.80E-15 | 4.65E-13 |
| L-Glutamic gamma-semialdehyde | up | C01165 | C5H9NO3 | 1.20 | 1.01 | 4.73E-05 | 0.00 |
| Methylacetate | up | C00163 | C3H6O2 | 1.19 | 1.02 | 7.44E-06 | 0.00 |
| Phthalic Acid | up | C01606 | C8H6O4 | 1.19 | 1.02 | 0.00 | 0.02 |
| Indole-3-glycol aldehyde | up | C03230 | C10H9NO2 | 1.18 | 1.02 | 0.01 | 0.05 |
| PS(18:1(9Z)/20:3(8Z,11Z,14Z)) | up | C02737 | C44H78NO10P | 1.18 | 1.04 | 0.00 | 0.01 |
| Propionic Acid | up | C00163 | C3H6O2 | 1.17 | 1.01 | 0.00 | 0.00 |
| 7,8-diaminopelargonate | up | C01037 | C9H20N2O2 | 1.15 | 1.03 | 2.01E-06 | 8.34E-06 |
| Trolamine | up | C06771 | C6H15NO3 | 1.14 | 1.01 | 3.84E-14 | 1.43E-12 |
| Succinic Acid | up | C00042 | C4H6O4 | 1.14 | 1.01 | 0.01 | 0.04 |
| Uridine 2',3'-cyclic phosphate | up | C02355 | C9H11N2O8P | 1.13 | 1.02 | 0.04 | 0.15 |
| Dihydroxyacetone | up | C00184 | C3H6O3 | 1.11 | 1.01 | 0.02 | 0.07 |
| Capric acid | up | C01571 | C10H20O2 | 1.09 | 1.01 | 8.37E-08 | 4.09E-06 |
| Alpha-D-Glucose | up | C00267 | C6H12O6 | 1.09 | 1.01 | 5.22E-05 | 0.00 |
| Gibberellin A9 | up | C11863 | C19H24O4 | 1.09 | 1.01 | 1.69E-05 | 0.00 |
| 9,10-DHOME | up | C14828 | C18H34O4 | 1.07 | 1.01 | 5.17E-10 | 4.47E-09 |
| Guanine | up | C00242 | C5H5N5O | 1.07 | 1.08 | 0.00 | 0.00 |
| 1-Methylxanthine | up | C16358 | C6H6N4O2 | 1.06 | 1.02 | 0.00 | 0.03 |
| KAPA | up | C01092 | C9H17NO3 | 1.06 | 1.02 | 4.48E-12 | 6.69E-11 |
| D-Ribose | up | C00121 | C5H10O5 | 1.06 | 1.01 | 0.00 | 0.02 |
| Floridin | up | C11754 | C19H17N3O4S2 | 1.06 | 1.01 | 5.34E-13 | 1.05E-11 |
| L-Rhamnulose | up | C00861 | C6H12O5 | 1.05 | 1.01 | 0.01 | 0.05 |
| 5-Thymidylic acid | up | C00364 | C10H15N2O8P | 1.05 | 1.01 | 0.01 | 0.07 |
| Leucodopachrome | up | C05604 | C9H9NO4 | 1.04 | 1.02 | 0.03 | 0.11 |
| Phenethylamine | up | C05332 | C8H11N | 1.04 | 1.02 | 2.41E-07 | 1.20E-06 |
| PC(18:4(6Z,9Z,12Z,15Z)/20:2(11Z,14Z)) | up | C00157 | C46H80NO8P | 1.03 | 1.03 | 0.01 | 0.01 |
| LysoPC(20:1(11Z)/0:0) | up | C04230 | C28H56NO7P | 1.02 | 1.03 | 0.01 | 0.02 |
| Methylmalonic Acid | up | C02170 | C4H6O4 | 1.01 | 1.01 | 0.01 | 0.04 |
| DUDP | up | C01346 | C9H14N2O11P2 | 1.01 | 1.01 | 0.01 | 0.06 |
| Mesobilirubinogen | down | C05790 | C33H44N4O6 | 2.60 | 0.90 | 0.03 | 0.12 |
| GlcCer(d18:1/20:0) | down | C01190 | C44H85NO8 | 2.50 | 0.96 | 2.51E-07 | 1.04E-05 |
| PE(18:0/18:1(11Z)) | down | C00350 | C41H80NO8P | 2.22 | 0.97 | 4.28E-06 | 0.00 |
| PE(18:0/20:1(11Z)) | down | C00350 | C43H84NO8P | 2.12 | 0.97 | 4.61E-06 | 0.00 |
| PE(P-18:0/20:4) | down | C00350 | C43H78NO7P | 2.05 | 0.98 | 8.30E-06 | 0.00 |
| PC(18:1(9Z)/P-16:0) | down | C00157 | C42H82NO7P | 1.93 | 0.98 | 9.40E-05 | 0.00 |
| Theobromine | down | C07480 | C7H8N4O2 | 1.80 | 0.90 | 0.01 | 0.01 |
| PS(20:1(11Z)/20:4(5Z,8Z,11Z,14Z)) | down | C02737 | C46H80NO10P | 1.78 | 0.98 | 6.16E-05 | 0.00 |
| PE(18:2(9Z,12Z)/18:0) | down | C00350 | C41H78NO8P | 1.74 | 0.98 | 0.00 | 0.01 |
| PC(22:4(7Z,10Z,13Z,16Z)/20:3(8Z,11Z,14Z)) | down | C00157 | C50H86NO8P | 1.68 | 0.98 | 6.20E-06 | 0.00 |
| Retinyl ester | down | C02075 | C20H30O2 | 1.65 | 0.96 | 4.84E-07 | 2.28E-06 |
| PS(20:3(8Z,11Z,14Z)/18:1(11Z)) | down | C02737 | C44H78NO10P | 1.60 | 0.96 | 0.02 | 0.08 |
| Fluorene | down | C07715 | C13H10 | 1.50 | 0.96 | 3.50E-08 | 2.09E-07 |
| P-Benzoquinone | down | C00472 | C6H4O2 | 1.49 | 0.91 | 0.00 | 0.00 |
| N-Formyl-L-glutamic acid | down | C01045 | C6H9NO5 | 1.45 | 0.97 | 5.17E-10 | 4.47E-09 |
| PS(18:2(9Z,12Z)/18:1(11Z)) | down | C02737 | C42H76NO10P | 1.39 | 0.96 | 0.04 | 0.13 |
| PC(18:1(9Z)/16:1(9Z)) | down | C00157 | C42H80NO8P | 1.37 | 0.97 | 3.05E-06 | 1.24E-05 |
| PS(18:1(11Z)/18:0) | down | C02737 | C42H80NO10P | 1.35 | 0.98 | 0.01 | 0.05 |
| Aldosterone | down | C01780 | C21H28O5 | 1.33 | 0.97 | 3.25E-09 | 2.36E-08 |
| Urobilinogen | down | C05791 | C33H42N4O6 | 1.30 | 0.94 | 0.05 | 0.08 |
| N-Choloylglycine | down | C01921 | C26H43NO6 | 1.27 | 0.94 | 0.04 | 0.06 |
| 2-Phenylacetamide | down | C02505 | C8H9NO | 1.25 | 0.99 | 2.17E-15 | 4.65E-13 |
| Linoelaidic acid | down | C01595 | C18H32O2 | 1.25 | 0.98 | 2.25E-13 | 5.34E-12 |
| PE(18:2(9Z,12Z)/16:0) | down | C00350 | C39H74NO8P | 1.24 | 0.98 | 0.02 | 0.08 |
| PE(16:0/20:4(5Z,8Z,11Z,14Z)) | down | C00350 | C41H74NO8P | 1.15 | 0.98 | 0.05 | 0.16 |
| Sphinganine 1-phosphate | down | C01120 | C18H40NO5P | 1.06 | 0.96 | 0.01 | 0.03 |
| PC(15:0/22:1(13Z)) | down | C00157 | C45H88NO8P | 1.06 | 0.97 | 7.75E-05 | 0.00 |
| SM(d18:0/16:1(9Z)) | down | C00550 | C39H79N2O6P | 1.04 | 0.99 | 0.00 | 0.00 |

**Table S3 Results of** **KEGG Topology Analysis**

| **First Category** | **Second Category** | **Pathway Description** | **Pathway_ID** | **Match_status** | **Impact**  **value** | **P** | **P_adj** |
| --- | --- | --- | --- | --- | --- | --- | --- |
| Metabolism | Lipid metabolism | Linoleic acid metabolism | map00591html | 2\|13 | 0.75 | 0.02 | 0.07 |
| Metabolism | Lipid metabolism | Glycerophospholipid metabolism | map00564html | 6\|52 | 0.23 | 0.00 | 0.00 |
| Metabolism | Biosynthesis of other secondary metabolites | Caffeine metabolism | map00232html | 2\|18 | 0.23 | 0.03 | 0.11 |
| Metabolism | Metabolism of cofactors and vitamins | Biotin metabolism | map00780html | 3\|24 | 0.17 | 0.01 | 0.03 |
| Metabolism | Global and overview maps | Nucleotide metabolism | map01232html | 5\|56 | 0.11 | 0.00 | 0.01 |
| Metabolism | Lipid metabolism | Sphingolipid metabolism | map00600html | 4\|23 | 0.11 | 0.00 | 0.00 |
| Metabolism | Carbohydrate metabolism | Citrate cycle (TCA cycle) | map00020html | 2\|20 | 0.09 | 0.04 | 0.12 |
| Metabolism | Nucleotide metabolism | Pyrimidine metabolism | map00240html | 6\|62 | 0.09 | 0.00 | 0.01 |
| Metabolism | Amino acid metabolism | Lysine degradation | map00310html | 4\|46 | 0.08 | 0.01 | 0.03 |
| Metabolism | Amino acid metabolism | Phenylalanine metabolism | map00360html | 5\|43 | 0.04 | 0.00 | 0.00 |
| Metabolism | Lipid metabolism | Steroid hormone biosynthesis | map00140html | 6\|89 | 0.03 | 0.00 | 0.01 |
| Metabolism | Carbohydrate metabolism | Propanoate metabolism | map00640html | 3\|36 | 0.01 | 0.02 | 0.07 |

First Category: Primary classification of metabolic pathways; Second Category: Secondary classification of metabolic pathways; Pathway ID: KEGG pathway ID; Match_status: Indicates the involvement of metabolites in the pathway, with the value to the left of the vertical bar representing the number of metabolites involved in this pathway from the current metabolite set, and the value to the right representing the total number of metabolites in the current pathway; Pathway Description: Name of the pathway; Impact_value: Comprehensive importance score of the pathway, with a maximum score of 1, calculated based on the relative position of metabolites in the pathway; P: Enrichment significance of metabolites in the pathway; P_adj: Adjusted P-value.

**Table S4 Prediction of SD based on Plasma metabolites**

| **Plasma metabolites** | **AUC** | **specificity** | **sensitivity** |
| --- | --- | --- | --- |
| Androst-5-ene-3beta,17beta-diol | 0.997 | 0.97 | 0.98 |
| Phytosphingosine | 0.986 | 0.97 | 0.94 |
| 4-hydroxysphinganine | 0.977 | 0.94 | 0.95 |
| PE(16:1(9Z)/P-18:1(11Z)) | 0.977 | 0.91 | 0.95 |
| 5-Aminopentanal | 0.963 | 0.84 | 0.97 |
| 5-Androstenediol | 0.962 | 0.94 | 0.89 |
| KAPA | 0.935 | 0.91 | 0.95 |
| Prenol | 0.9 | 0.84 | 0.81 |

**Table S5 Top 20 Important Endogenous Differential Metabolites Selected by Random Forest**

| **Metabolites** | **Importance*** |
| --- | --- |
| Androst-5-ene-3beta,17beta-diol | 0.075064661 |
| Phytosphingosine | 0.059022923 |
| 4-hydroxysphinganine | 0.053659621 |
| 5-Androstenediol | 0.050222718 |
| KAPA | 0.03885017 |
| 5-Aminopentanal | 0.036245982 |
| Prenol | 0.028759213 |
| PE(16:1(9Z)/P-18:1(11Z)) | 0.027642897 |
| Aldosterone | 0.017140319 |
| PE(18:0/20:1(11Z)) | 0.010173727 |
| L-Pipecolic acid | 0.010118196 |
| 7,8-diaminopelargonate | 0.00970434 |
| N-Formyl-L-glutamic acid | 0.009516768 |
| Adenine | 0.00844617 |
| 9,10-DHOME | 0.007894038 |
| Isocitrate | 0.006290203 |
| 2,3,4-Trihydroxybutanoic acid | 0.005882985 |
| PG(i-12:0/18:2(9Z,11Z)) | 0.005402872 |
| PC(15:0/22:1(13Z)) | 0.00500814 |
| N-Acetyl-L-phenylalanine | 0.004873897 |

^*^Importance represents the significance value corresponding to each metabolite, with higher values indicating greater importance of the metabolite.
